# A First Principles study on Boron-doped Graphene decorated by Ni-Ti-Mg atoms for Enhanced Hydrogen Storage Performance

**DOI:** 10.1038/srep16797

**Published:** 2015-11-18

**Authors:** Santhanamoorthi Nachimuthu, Po-Jung Lai, Ermias Girma Leggesse, Jyh-Chiang Jiang

**Affiliations:** 1 Department of Chemical Engineering, National Taiwan University of Science and Technology, Taipei 106, Taiwan, R.O.C

## Abstract

We proposed a new solid state material for hydrogen storage, which consists of a combination of both transition and alkaline earth metal atoms decorating a boron-doped graphene surface. Hydrogen adsorption and desorption on this material was investigated using density functional theory calculations. We find that the diffusion barriers for H atom migration and desorption energies are lower than for the previously designed mediums and the proposed medium can reach the gravimetric capacity of ~6.5 wt % hydrogen, which is much higher than the DOE target for the year 2015. Molecular Dynamics simulations show that metal atoms are stably adsorbed on the B doped graphene surface without clustering, which will enhance the hydrogen storage capacity.

Hydrogen is one of the promising alternative energy sources for future generations due to its applications in hydrogen fuel-cell powered vehicles^1^,^2^,^3^. However, designing solid state material for hydrogen storage with high gravimetric and volumetric densities near ambient thermodynamic conditions is a challenging task^4^,^5^. In recent years, many systems have been proposed to store hydrogen, but none of them has achieved the US Department of Energy (DOE) target for 2015. Therefore, it is essential to explore new materials with desired properties for efficient and economic ways of storing hydrogen.

Carbon-based materials such as graphene and carbon nanotubes have been designed for hydrogen storage due to their large surface area, light weight and tunable properties^6^,^7^,^8^,^9^. However, the interaction of carbon with hydrogen is only through weak van der Waals forces and hence their binding energies are very low, resulted in decreasing the storage capacity^10^,^11^. In recent years, carbon-based nanostructures decorated with alkaline earth metal atoms (AEM)^12^,^13^,^14^ and transition metal atoms (TM)^15^,^16^,^17^,^18^,^19^,^20^,^21^,^22^,^23^ are proposed to increase the interaction between the hydrogen molecules and the host materials. The decoration by TM atoms increases the binding ability of hydrogen, because of the hybridization of H_2_ σ or σ* orbitals with *d*-orbitals of TM atoms (the so-called the interaction typical of Kubas complexes)^24^,^25^,^26^. However, because of the large cohesive energy, TM atoms form a cluster, which decrease the hydrogen storage capacity^27^,^28^. The recent studies suggest that the doping of Boron (B) atom on graphene can prevent the clustering of the isolated TM atoms and increase the hydrogen storage capacity^29^,^30^,^31^,^32^. Also, Louie *et al*.^33^ reported that the B-doping enhanced the binding energy of Ca atom and it suppresses the aggregation of Ca atoms.

Most of the previous studies describe the hydrogen storage medium decorated by either AEM or TM atoms on the graphene surface. However, in the former case, the adsorption energy of hydrogen is too small because of weak interaction and hence the number of adsorbed hydrogen molecules is less, whereas in the latter case, desorption of hydrogen is challenging because of its larger adsorption energy. Recently, we decorated a B-doped graphene sheet by three different TM atoms (Ni-Pd-Co) and reported their hydrogen storage efficiency^34^. But, those TM atoms are heavy and their wt.% is also comparatively low. Hence, in this present study we propose a new strategy to enhance both hydrogen adsorption/desorption at ambient conditions and to reach the gravimetric goal of DOE. Here we considered combination of both TM and AEM atoms to decorate the B-doped graphene (BDG) surface and investigate the adsorption and desorption of hydrogen using first-principles calculations. To the best of our knowledge, the combination of TM and AEM atoms for hydrogen storage application has not yet been reported.

## Results and Discussion

As reported in our earlier study and previous studies^34^,^35^, the decoration of graphene by metal atoms improves the hydrogen storage capacity. In order to increase the wt.% from our previously considered TM atoms, here we considered Ni and Ti of TM atoms in one end to adsorb the hydrogen and Mg of AEM atom for another end to desorb. We have carefully chosen these metals because of its strong, medium and weak hydrogen adsorption energies. The calculated hydrogen adsorption energies for TM atoms (Ni and Ti) are −0.92 and −0.52 eV, and for Mg it is nearly zero (−0.01 eV)^34^. More importantly the molecular weight of these atoms is much smaller than the previously considered atoms. These three metal atoms (Ni-Ti-Mg) are used to decorate the BDG surface and we investigate the hydrogen storage capacity by the spillover mechanism. Previous studies reported that the hydrogen storage capacity can be improved by means of the spillover mechanism^36^,^37^,^38^,^39^,^40^. We expect that the relatively low hydrogen adsorption energy of Mg will contribute a low hydrogen desorption energy and hence, hydrogen can desorb at room temperature, which is essential for the practical applications.

In order to find the possible binding sites of these metals on the BDG surface, we considered three different sites such as 1) hollow site, (the center of the ring) 2) bridge site (in between the two atoms) and 3) top site (top of the atoms) for the adsorption of a single metal on BDG surface. The binding energy (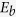) of these metals with surface is calculated using 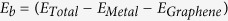, where 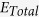, 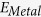 and 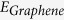 are the energies of total system, Metal atoms and B-doped graphene surface, respectively. These calculations indicate that these three metal atoms Ni, Ti and Mg, are most stably adsorbed on the surface by hollow sites with binding energies of −3.53, −2.10 and −1.49 eV, respectively. It is found that the calculated binding energies for Ni and Ti atoms on BDG surface are larger than the pristine graphene layer^41^, which indicate an increase in the stability of the materials after B-doping. As a first step, we considered trimers of each of these three metal atoms adsorbed on the BDG surface via three different sites such as, top of the boron atom (Site A), top of the carbon atom which is surrounded by three boron atoms (Site B) and top of the carbon atom which is surrounded by two boron atoms (Site C). Here, we determined the trimer of each metal atom that would increase the wt.%. of hydrogen storage and also the distance between the two different types of metal trimer in surface that would be most favorable for hydrogen diffusion. The top views of the three possible binding sites (Sites A, B and C) for Ni metal trimer adsorbed on BDG surface are shown in Figure 1S of supplementary material. The calculated binding energies for different sites are given in Table 1. As can be seen from this table, Site A and Site B have strong binding energies for both the TM atoms and AEM atom, respectively. It was reported that strong binding energies of the metal atoms with the surface can prevent clustering, because of strong covalent bonds between the metal atoms and the surface^42^. Therefore, we considered the geometries with strong metal surface binding energies for the further calculations.

To investigate the hydrogen storage capacity, we gradually increased the number of adsorbed H_2_ molecules on the Ni and Ti atom decorated BDG surface and calculated the adsorption energy values using the formula, 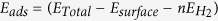. The calculated total adsorption energy and average adsorption energy per H_2_ molecule are given in Table 2. We found that the maximum of nine H_2_ molecules can be adsorbed on both the Ni and Ti decorated BDG surface with an average binding energy of −0.43 and −0.41 eV per H_2_ molecule, respectively. It has been observed that beyond this maximum value, the calculated binding energy values decreases abruptly, which may lead to decrease the stability of the system. Our calculation indicates that among the nine H_2_ molecules, the first four are adsorbed via chemisorption, i.e., dissociated into 8 hydrogen atoms on the both Ni and Ti decorated BDG surface and further adsorption follows as a physisorption (i.e., molecular form).

It is well known that the hydrogen spillover is the diffusion of dissociated hydrogen atoms adsorbed on one metal site to the nearest metal of a different type on the decorated surface. Here, hydrogen diffusion must occur according to the following steps: (1) The Ni-decorated BDG surface adsorbs H_2_ and diffuses to nearest metal Ti; and (2) the Ti-decorated BDG surface diffuses hydrogen to the nearest Mg metal. Here, we considered the atomic diffusion of H from one metal to another metal (i.e., from Ni to Ti and Ti to Mg) and also we considered the effect of hydrogen coverage in each case (i.e, minimum and maximum hydrogen adsorption). In order to accommodate two kinds of metals, the BDG surface is extended to 6 × 3 unit cell from 3 × 3 and we optimize the surface to ensure different kinds of metal atoms will not attract each other to form clusters.

**Figure 1:**
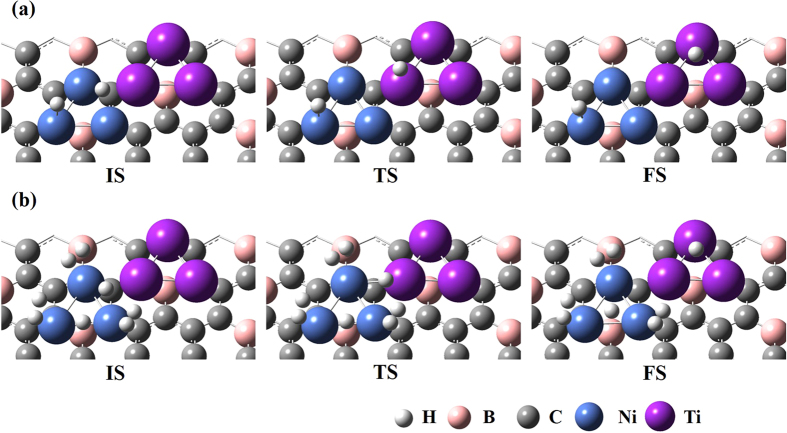
Side view of initial (IS), Transition (TS) and Final state (FS) geometries for the diffusion of one H atom from Ni to Ti in (a) minimum and (b) maximum hydrogen coverage.

**Figure 2:**
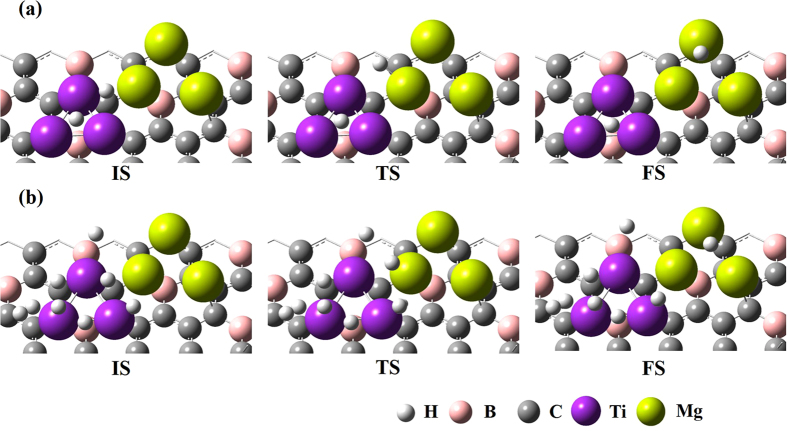
Side view of initial (IS), Transition (TS) and Final state (FS) geometries for the diffusion of one H atom from Ti to Mg in (a) minimum and (b) maximum hydrogen coverage.

First, we considered one hydrogen molecule adsorbed (via chemisorption) on Ni trimer decorated on the BDG surface and the diffusion of one hydrogen atom to the nearest Ti trimer. Figure 1 shows the corresponding initial state (IS), transition state (TS) and final state (FS) for the atomic H diffusion from Ni to Ti for both minimum and maximum hydrogen coverages for the chemisorption case. The diffusion barriers and the minimum energy path (MEP) for all the cases considered in this study are calculated using NEB method^43^,^44^ and the values are given in the Table 1S of supplementary material. We found that the distance between the two H atoms is 2.42 Å in the initial state and the calculated activation energy for the one H atom diffusion from Ni trimer to Ti is 0.65 eV. The calculated reaction energy is exothermic by −0.67 eV. We included zero-point energy corrections in all of our calculations. In the maximum coverage, (i.e,4 H_2_ adsorbed on Ni via chemisorption) among 8 H atoms, we considered diffusion of the H atom that had the shortest distance to the nearest Ti trimer and the calculated activation barrier is 0.41 eV and the reaction energy is exothermic by −1.53 eV. Second we considered the diffusion of one H atom from Ti to Mg both in minimum and maximum coverages. The optimized geometries of IS, TS, and FS for the diffusion path of the H atom from Ti to Mg are illustrated in Fig. 2. The activation barrier for the migration of one H atom from Ti to Mg in the case of minimum coverage is found to be 0.67 eV and the calculated reaction energy is endothermic by 0.63 eV. The calculated diffusion barrier and the reaction energy for the migration of one H atom in the case of maximum hydrogen coverage (i.e.,4 H_2_ adsorbed on Ti via Chemisorption) is 0.32 and 0.23 eV, respectively. From Table 1S, we found that the diffusion barrier decreases while increasing the hydrogen coverage which is in agreement with our previous study^34^. Further, the calculated diffusion barriers for the migration of the H atom in this combination of both TM and AEM atoms is comparatively lower than our recent report on a BDG surface decorated by TM atoms alone^34^ and also other studies^32^,^40^. It has been observed that the replacing the previously reported metal atoms (i.e., Pd by Ti and Co by Mg) significantly reduces the diffusion barriers both in the minimum and maximum hydrogen coverages. Further, we expect molecular (H_2_) diffusion from one metal to another one in the case of physisorption. We found in our previous study^34^ that the diffusion barriers for H_2_ migration on metal atoms decorated BDG are much lower than those for atomic diffusion. Similarly, here also we believe that the diffusion barriers for H_2_ migration should be less than atomic diffusion.

Table 3 shows the calculated desorption energies (negative of the adsorption energy) of the H_2_ molecules from Mg decorated on the BDG surface. The desorption energy for the minimum coverage, i.e., only one H_2_ molecule, is found to be 0.52 eV and it decreases with increasing the hydrogen coverages. We found that a maximum of six H_2_ molecules can adsorb on the Mg trimer. In the maximum coverage, the desorption energy per H_2_ molecule is only 0.18 eV, which is close to the required energy for reversible storage. Considered AEM atom Mg is lighter than the previously used TM atom Co and also the calculated desorption energies are very low compared to our previous study^34^ and hence it is easy to desorb the diffused H from the terminal part.

**Figure 3:**
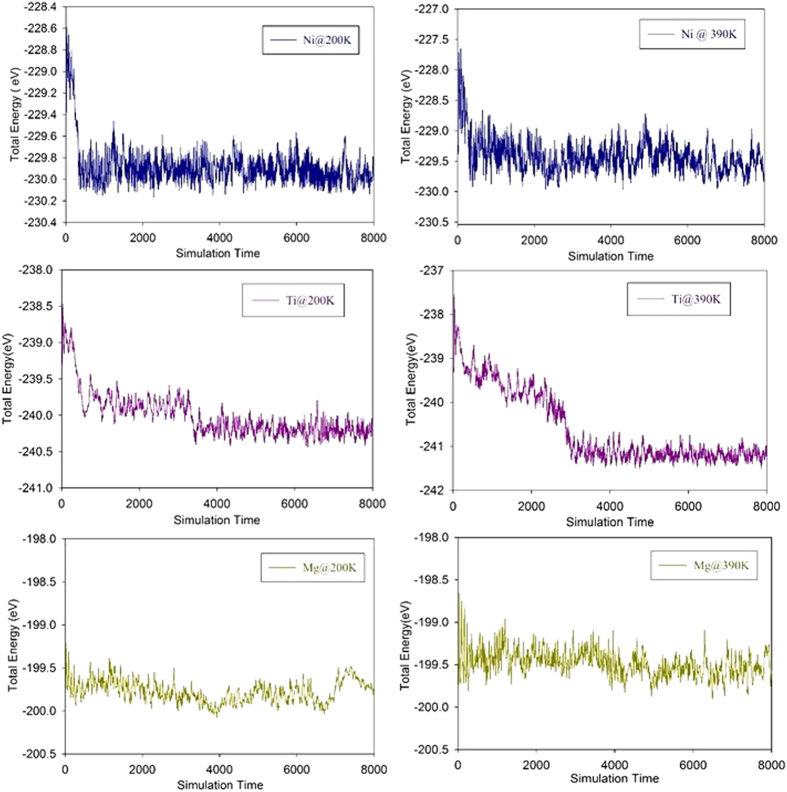
Fluctuations of total energy as a function of simulation time for different metal atoms decorated B-doped Graphene surface in molecular dynamics simulations at different temperatures (200 and 390 K).

Further, in order to confirm that the proposed hydrogen storage medium is thermodynamically stable, we performed molecular dynamics (MD) simulations using the constant particle number, volume, temperature (NVT) ensemble on these metal decorated B-doped graphene surface in the case of maximum hydrogen adsorption. The suggested temperature for H_2_ delivery by the DOE is in the range of 233−393 K. Hence, we performed MD simulations for maximum number of H_2_ molecules adsorbed on Ni, Ti and Mg decorated boron-doped graphene surfaces at three different temperatures (200, 300, and 390 K). The equilibrated geometries of the different metal atoms decorated BDG surfaces at higher temperature are shown in Figure 2S of supplementary information. The fluctuations of total energy as a function of simulation time at different temperatures (200 and 390 K) are shown in Fig. 3 and for 300 K given in Figure 3S of Supplementary information. As can be clearly seen from those figures, there is no structural deformation of the proposed medium even at higher temperatures, which clearly confirms that the considered metal atoms dispersed uniformly and stably adsorbed on the B doped graphene surface without clustering. Our MD results show that H_2_ molecules are stably adsorbed on the metal atoms at low temperature and all adsorbed H_2_ molecules start to desorb from the metal atoms with increasing the temperature and are completely desorbed at higher temperatures.

In summary, we have designed a new and efficient solid state material consisting of a combination of both TM and AEM atoms which have potentially a good reversibility of hydrogen uptake and release. These TM and AEM atoms decorated BDG surface can have the maximum hydrogen gravimetric capacity of 6.4% for double-sided adsorptions, which is much higher than the DOE new target of 5.5% for the year 2015^45^. Molecular Dynamics simulations were also carried out to test the stability of the designed medium at different temperatures. Results show that the clustering of metal atoms is suppressed in the proposed medium and it is stable even at larger temperature. Hence, the designed medium will be a good candidate for the efficient hydrogen storage at ambient conditions and we hope this theoretical study can guide experimental works on the designing of solid-state media for hydrogen storage based on carbon nanomaterials.

## Methods

All the DFT calculations and first-principles molecular dynamics (MD) simulations were carried out using the Vienna ab initio simulation package (VASP)^46^ with a projector-augmented-wave (PAW) method^47^. The exchange-correlation energy functional in the generalized gradient approximation (GGA) of Perdew and Wang (PW91)^48^ was used. The graphene used in our simulation consists of a 3 × 3 supercell of dimension 7.60 × 7.60 × 13.00 (Å) with a vacuum thickness of 13 Å to avoid interlayer interaction. Further, the possible atomic arrangement for B-doped graphene (BC_5_) is based on previous experimental results^49^. The sampling of the Brillouin zone is done for 3 × 3 and 6 × 3 super cells with 5 × 5 × 1 and 2 × 5 × 1 Monkhorst-Pack k-point grids, respectively. Spin−polarized calculations have been performed in all the cases. The cutoff energy was set to 500 eV for a fully relaxed geometry optimization. All the structures were optimized until the total energy converged to less than 10^−5^ eV per atom and the maximum force converged to lower than 0.02 eVÅ^−1^. The possible reaction pathway for hydrogen atom diffusion from one metal to the nearest metal has been considered and the nudged elastic band (NEB) method^43^,^44^ was used to estimate the transition structures and diffusion barriers. Zero-point energy (ZPE) corrections have been included for all the diffusion barrier calculations, which is calculated as: 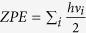, where h is Planck’s constant and 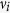
(Hz) is runs over all computed frequencies. In order to reduce the computational cost, we considered only metal and hydrogen atoms for the frequency calculations. Further, in order to confirm the thermal stability of these metal decorated B-doped surface, we performed ab initio molecular dynamics as implemented in VASP. The energy optimized structures were chosen as an initial configurations where Γ points are used for Brillouin zone integration throughout the simulations. The velocity Verlet algorithm^50^ was used to integrate Newton’s equation of motion with the simulation time step of 0.5 fs for 4 ps. The Nosé-Hoover thermostat^51^ method was used to control the different temperatures during the simulations.

## Additional Information

**How to cite this article**: Nachimuthu, S. *et al*. A First Principles study on Boron-doped Graphene decorated by Ni-Ti-Mg atoms for Enhanced Hydrogen Storage Performance. *Sci. Rep*. **5**, 16797; doi: 10.1038/srep16797 (2015).

## Supplementary Material

Supplementary Information

## Figures and Tables

**Table 1:** The calculated binding energies (E_b_ in eV) of metal trimer on graphene layer with three possible binding sites and nearest distances of metal to graphene surface (*d*_M-G_ in Å) and metal to metal (*d*_M-M_ in Å).

Metal	Binding Site	E_b_	*d*_M-G_	*d*_M-M_
	Site A	−4.99	1.54	2.40
Ni	Site B	−4.57	1.55	2.43
	Site C	−4.69	1.54	2.41
	Site A	−8.43	1.61	2.53
Ti	Site B	−8.26	1.76	2.52
	Site C	−8.37	1.71	2.55
	Site A	−3.62	1.79	3.03
Mg	Site B	−3.85	1.91	3.08
	Site C	−3.82	1.90	3.12

**Table 2:** The calculated total hydrogen adsorption energies (E_ads_ in eV) and adsorption energies per H_2_ molecule (E_ads_/H_2_ in eV) for Ni and Ti trimer decorated BDG surface.

Number of H_2_	Ni		Ti	
	E_ads_	E_ads_/H_2_	E_ads_	E_ads_/H_2_
1	−1.90	−1.90	−2.02	−2.02
2	−2.51	−1.25	−3.11	−1.56
3	−3.00	−1.00	−3.13	−1.04
4	−3.71	−0.93	−3.12	−0.78
5	−3.72	−0.74	−3.62	−0.72
6	−3.74	−0.62	−3.67	−0.61
7	−3.77	−0.54	−3.67	−0.52
8	−3.81	−0.48	−3.66	−0.46
9	−3.83	−0.43	−3.69	−0.41

**Table 3:** The calculated desorption energies (−E_ads_ in eV) and desorption energies per H_2_ molecule (−E_ads_/H_2_ in eV) for the hydrogen molecules adsorbed on Mg trimer decorated BDG surface.

Number of H_2_	Mg	
	−E_ads_	−E_ads_/H_2_
1	0.52	0.52
2	0.77	0.38
3	1.06	0.35
4	1.06	0.27
5	1.08	0.22
6	1.10	0.18
