# Supplementary material for: A First Principles study on Boron-doped Graphene decorated by Ni-Ti-Mg atoms for Enhanced Hydrogen Storage Performance
Source: Sci Rep. 2015 Nov 18;5:16797. doi: 10.1038/srep16797 (PMC4649468; doi:10.1038/srep16797)
Supplement: Supplementary Information [file srep16797-s1.doc]

**Supplementary Information**

**A First Principles study on Boron-doped Graphene decorated by Ni-Ti-Mg atoms for Enhanced Hydrogen Storage Performance**

Santhanamoorthi Nachimuthu, Po-Jung Lai, Ermias Girma Leggesse and Jyh-Chiang Jiang[[1]](#footnote-2)

Department of Chemical Engineering, National Taiwan University of Science and Technology, Taipei 106, Taiwan, R.O.C.

Table 1S. The calculated diffusion barriers (^‡^E in eV), reaction energies (ΔE in eV) and corresponding imaginary frequencies (IMF in cm^-1^) for the H atom diffusion from Ni to Ti and Ti to Mg in both minimum and maximum hydrogen coverage’s.

| **Parameter** | **Ni-Ti** | | **Ti-Mg** | |
| --- | --- | --- | --- | --- |
|  | **Minimum Coverage(H_2_)** | **Maximum Coverage(4 H_2_)** | **Minimum Coverage(H_2_)** | **Maximum Coverage(4 H_2_)** |
| **^‡^E** | 0.71 | 0.52 | 0.73 | 0.36 |
| **^‡^E_ZPE_** | 0.65 | 0.41 | 0.67 | 0.32 |
| **ΔE** | -0.66 | -1.45 | 0.64 | 0.31 |
| **ΔE_ZPE_** | -0.67 | -1.53 | 0.63 | 0.23 |
| **IMF** | 640*i* | 577*i* | 643*i* | 443*i* |

ZPE denotes the energies after Zero point Energy Correction

**
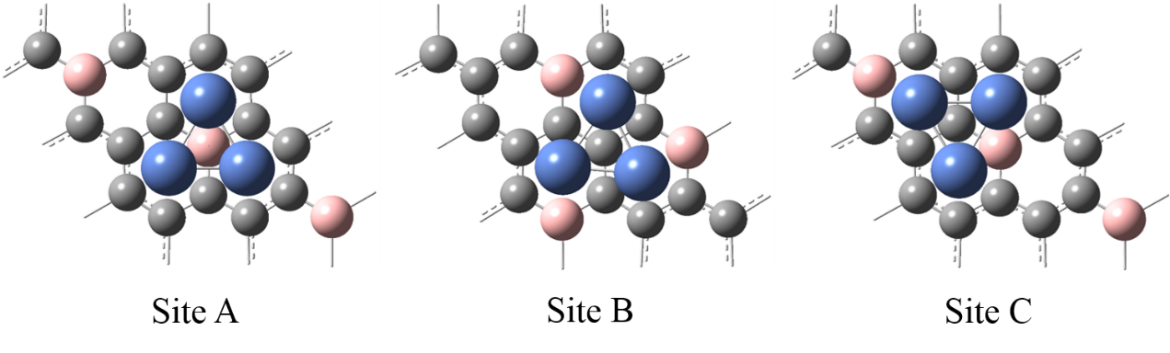
**

**Figure 1S.** The top view of three possible binding sites for Ni metal trimer adsorbed on BDG surface.


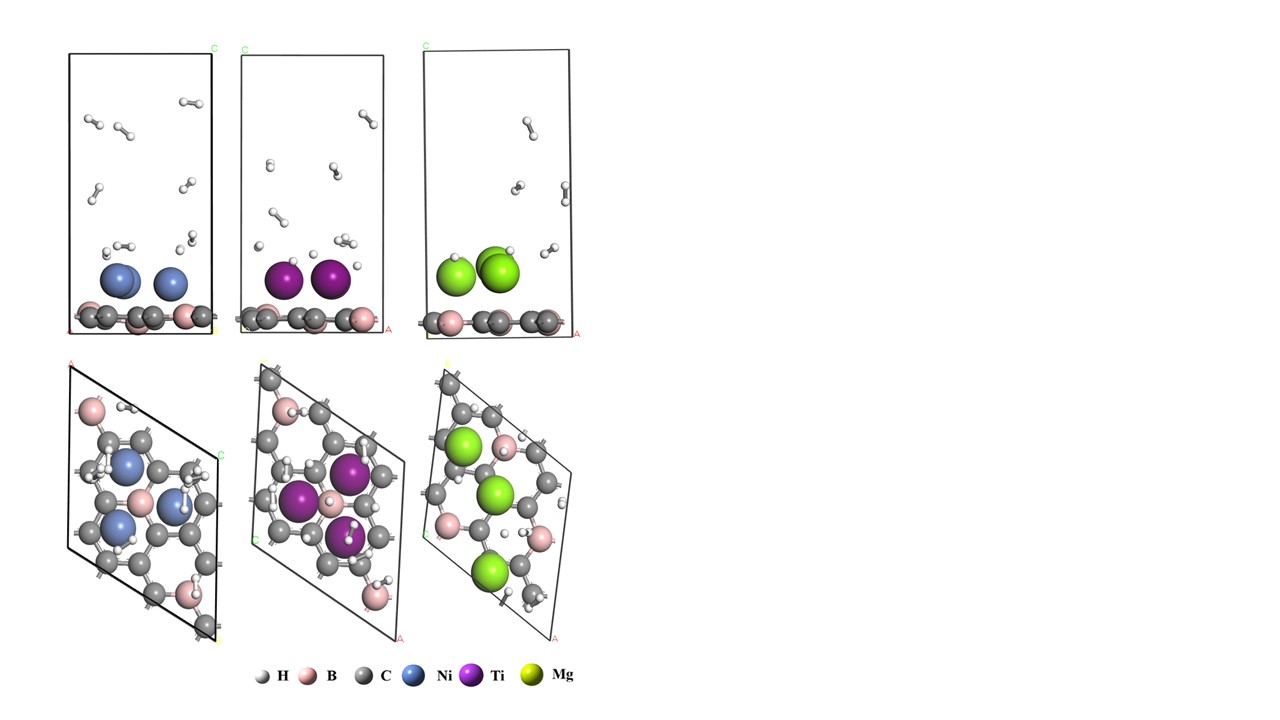


(b)


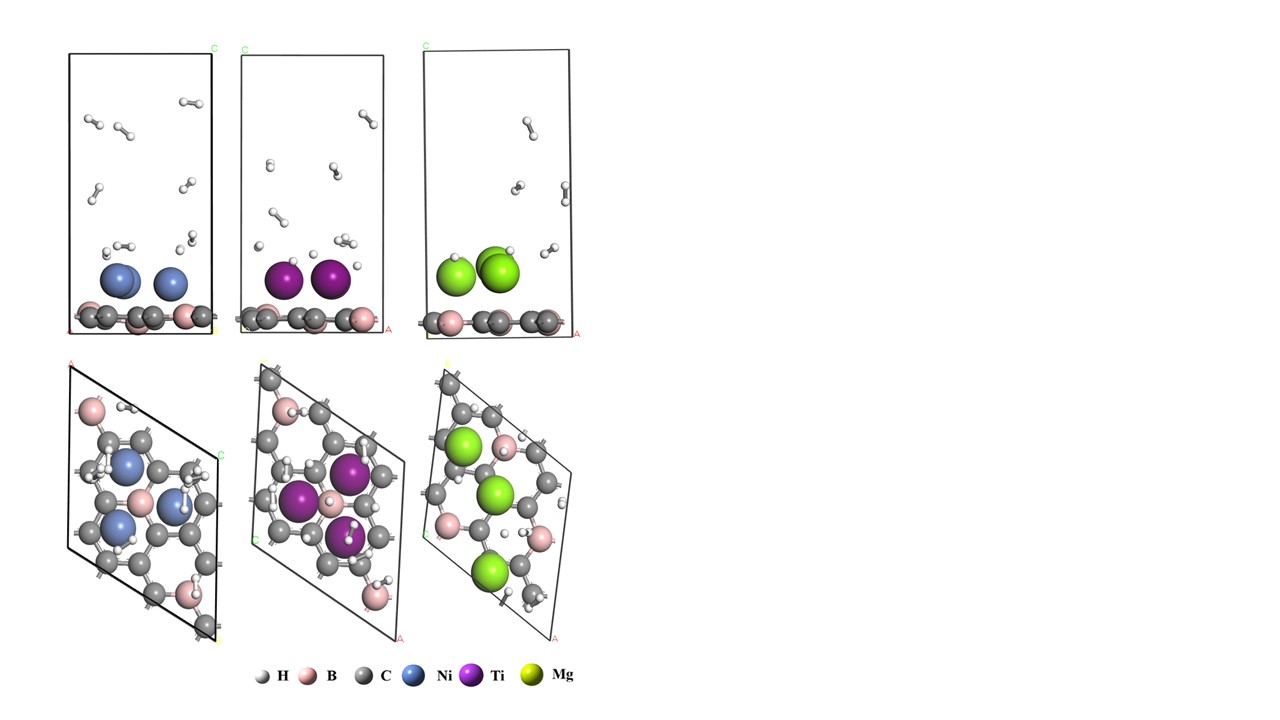


**Figure 2S.** The equilibrated structures of different metal atoms (Ni, Ti and Mg) decorated Boron doped graphene surface after MD simulations at 390K. ((a) Side view and (b) the top view of the (a))


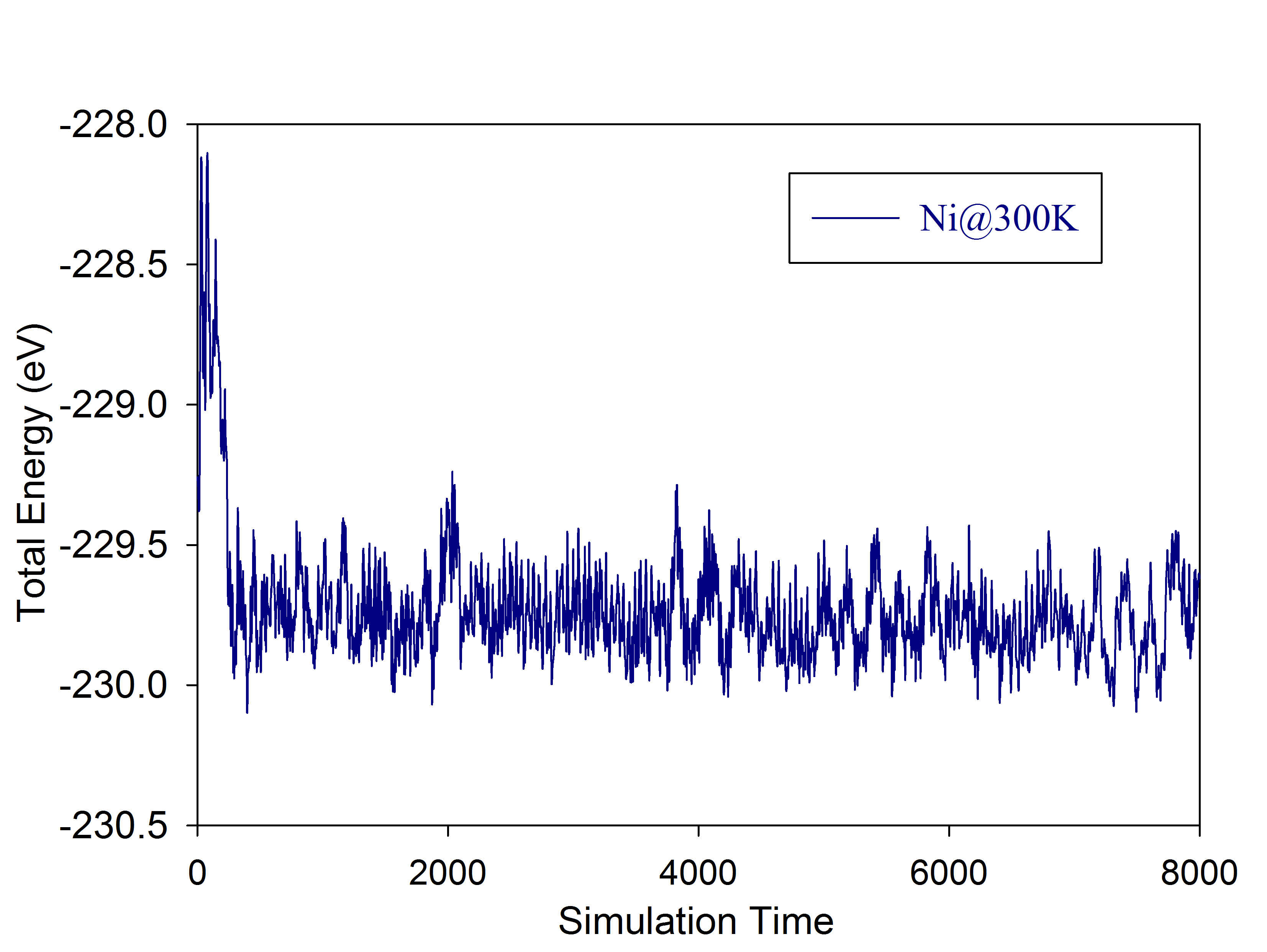


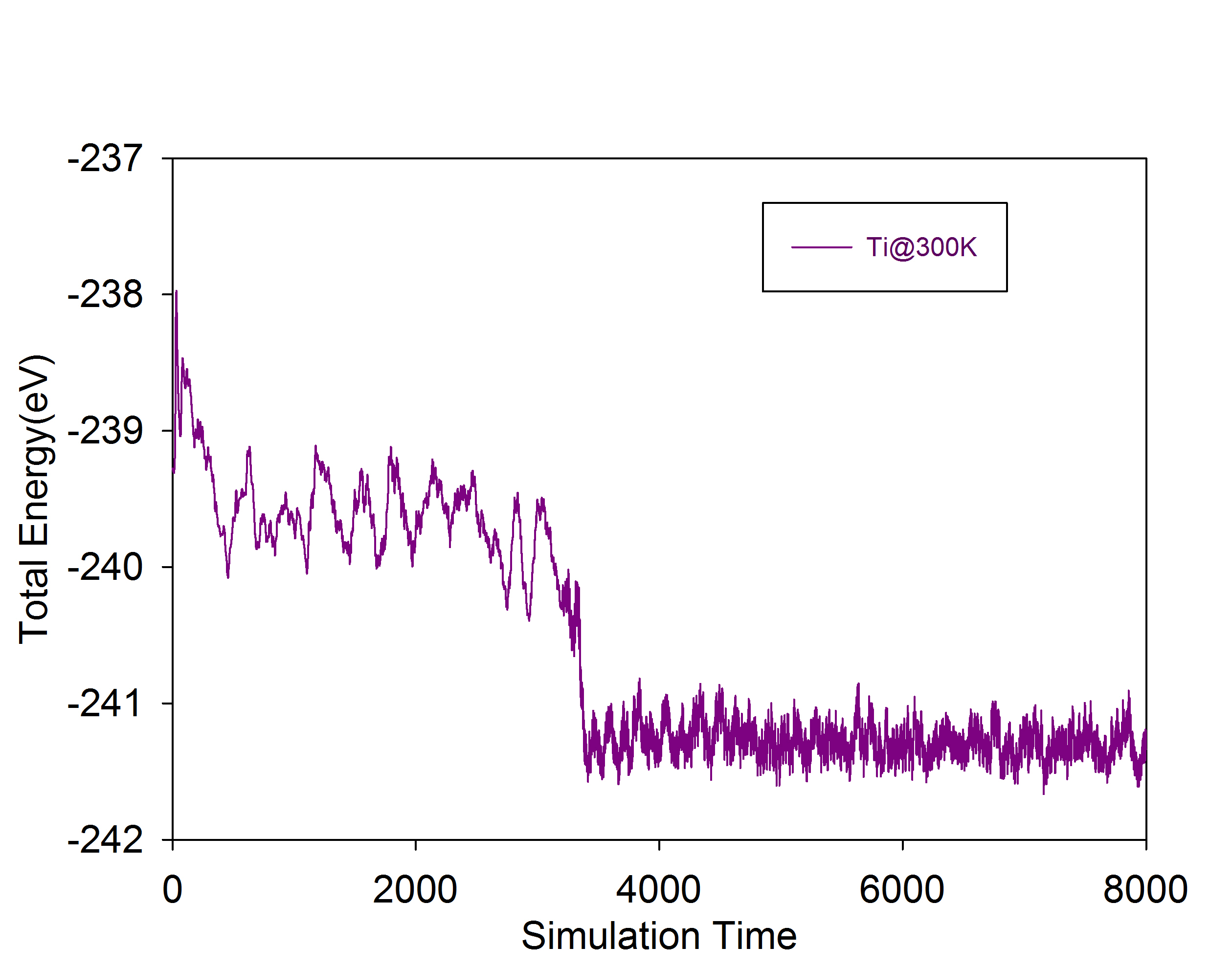


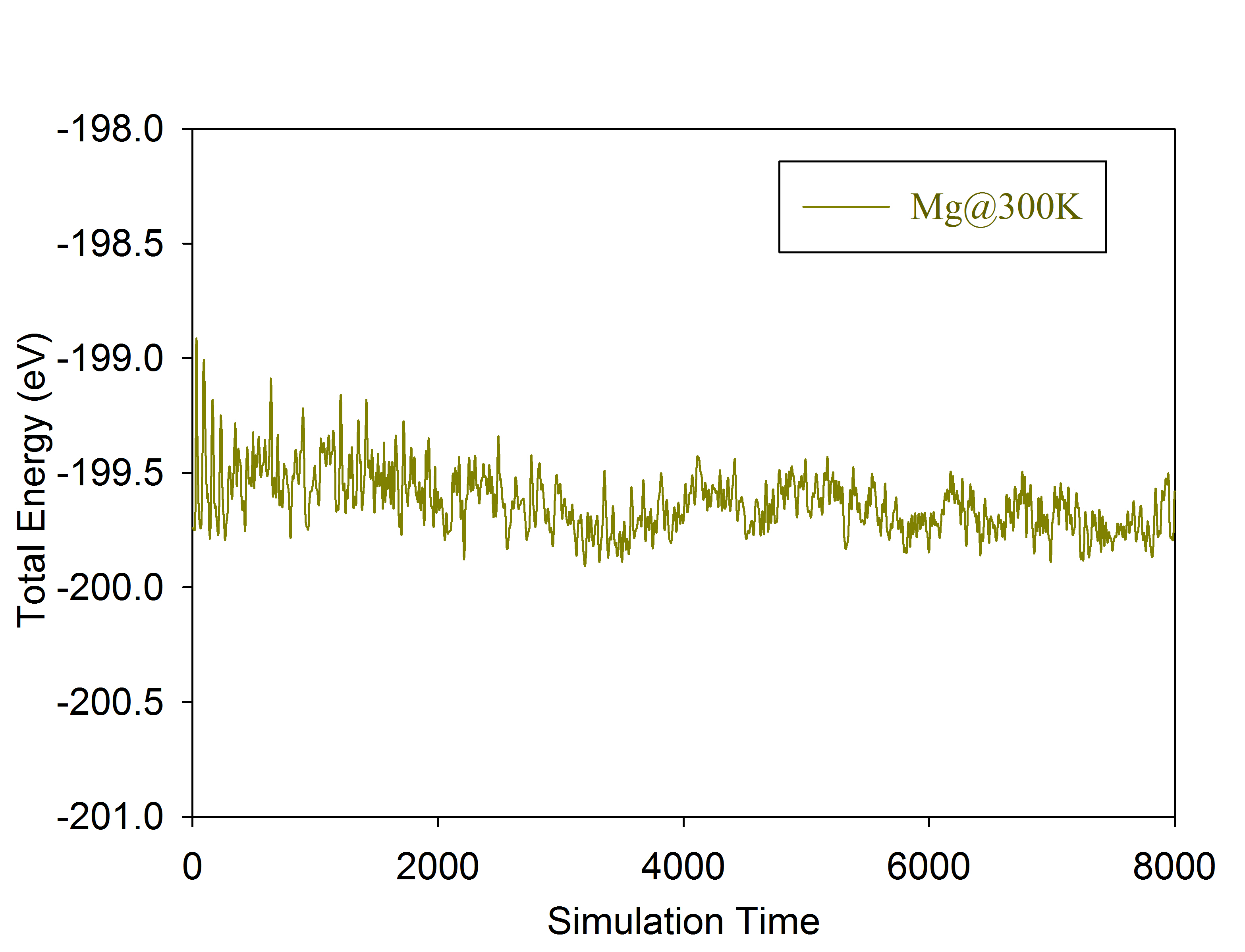


**Figure 3S.** Fluctuations of total energy as a function of simulation time for different metal atoms decorated B-doped Graphene surface in molecular dynamics simulations at 300K temperature.

1. Corresponding author Tel.: +886-2-27376653. Fax: +886-2-27376644

   E-mail address: [jcjiang@mail.ntust.edu.tw](mailto:jcjiang@mail.ntust.edu.tw) [↑](#footnote-ref-2)
